# Short-term outcomes of colorectal cancer surgery in older patients: a novel nomogram predicting postoperative morbi-mortality

**DOI:** 10.1007/s00423-022-02688-1

**Published:** 2022-09-21

**Authors:** David Moro-Valdezate, José Martín-Arévalo, Óscar Ferro-Echevarría, Vicente Pla-Martí, Stephanie García-Botello, Leticia Pérez-Santiago, Ricardo Gadea-Mateo, Noelia Tarazona, Desamparados Roda, Susana Roselló-Keränen, Alejandro Espí-Macías

**Affiliations:** 1 Colorectal Surgery Unit, Department of General and Digestive Surgery, INCLIVA Biomedical Research Institute, Hospital Clínico Universitario de Valencia, Av. Blasco Ibáñez, 17, 46010 Valencia, Spain; 2 Department of Surgery, University of Valencia, Valencia, Spain; 3 Department of Medical Oncology, INCLIVA Biomedical Research Institute, Hospital Clínico Universitario de Valencia, Valencia, Spain

**Keywords:** Colorectal cancer, Older patients, Morbidity, Mortality, Risk factors, Nomograms

## Abstract

**Purpose:**

To analyze short-term outcomes of curative-intent cancer surgery in all adult patients diagnosed with colorectal cancer undergoing surgery from January 2010 to December 2019 and determine risk factors for postoperative complications and mortality.

**Methods:**

Retrospective study conducted at a single tertiary university institution. Patients were stratified by age into two groups: < 75 years and ≥ 75 years. Primary outcome was the influence of age on 30-day complications and mortality. Independent risk factors for postoperative adverse events or mortality were analyzed, and two novel nomograms were constructed.

**Results:**

Of the 1486 patients included, 580 were older (≥ 75 years). Older subjects presented more comorbidities and tumors were located mainly in right colon (45.7%). After matching, no between-group differences in surgical postoperative complications were observed. The 30-day mortality rate was 5.3% for the older and 0.8% for the non-older group (*p* < 0.001). In multivariable analysis, the independent risk factors for postoperative complications were peripheral vascular disease, chronic pulmonary disease, severe liver disease, postoperative transfusion, and surgical approach. Independent risk factors for 30-day mortality were age ≥ 80 years, cerebrovascular disease, severe liver disease, and postoperative transfusion. The model was internally and externally validated, showing high accuracy.

**Conclusion:**

Patients aged ≥ 75 years had similar postoperative complications but higher 30-day mortality than their younger counterparts. Patients with peripheral vascular disease, chronic pulmonary disease, or severe liver disease should be informed of higher postoperative complications. But patients aged ≥ 80 suffering cerebrovascular disease, severe liver disease, or needing postoperative transfusion should be warned of significantly increased risk of postoperative mortality.

**Supplementary information:**

The online version contains supplementary material available at 10.1007/s00423-022-02688-1.

## Introduction

Against the background of the progressive ageing of the European population, currently 9.84% of the Spanish population is ≥ 75 years old, and Spain is estimated to become one of the longest-living countries in Europe within 40 years [1]. This trend has serious implications, as older patients needing a surgical resection for colorectal cancer (CRC) are more likely than the non-older to present with medical and surgical postoperative complications [2, 3], probably due to the fact that comorbidities are more frequent and serious in older age patients [4–6]. Surgeons are therefore faced with difficult decisions when managing increasingly older patients. It is estimated that postoperative adverse outcomes in this patient subgroup could be substantial, ranging from 6 to 50% [7–12]. Higher postoperative mortality rates of up to 20% have also been reported in older patients, mainly during the first 30 days after surgery [4, 5, 9, 11–16].

However, age may not be the only variable influencing surgical outcomes in the older. Indeed, some studies found no significant differences in rates of postoperative complications, reoperations, or mortality between patients over or under 80 years of age [6, 13, 17–19]. Several factors have been identified that could increase the risk of postoperative adverse events in older patients: comorbidities, male sex, tumor location, operation time, open surgery, and emergent surgery. Interestingly, age has not appeared as a factor increasing postoperative complications or mortality rate [12, 14, 17–21]. Some authors have reported comorbidities as the strongest predictors of postoperative complications in aged patients [8]. Preoperative identification of predictors of surgical complications in older patients could be useful for implementing additional optimization bundles before major surgery.

The aim of this study was to assess postoperative outcomes after curative-intent oncologic surgery for CRC and determine independent risk factors for complications or mortality during the first 30 post-surgery days.

## Materials and methods

### Study design and setting

This observational study included all adult patients diagnosed with CCR from January 2010 to December 2019 at the Colorectal Surgery Department in a tertiary university institution (University Clinic Hospital of Valencia, Spain). The STROBE guidelines were followed [22]. Tumors were staged according to the 8th edition of the American Joint Committee on Cancer classification. The inclusion criteria were age over 18 years, histological diagnosis of stages I-III colon or rectal adenocarcinoma, indication for elective oncological surgery with curative intent and minimum follow-up of 1 year. Exclusion criteria were appendicular tumor and local rectal excision. Patients were stratified according to significant age-specific cut-off points for this series. The older cohort was matched to the young cohort by propensity score analysis to obtain two comparable patient groups.

### Data source and study variables

Patient data were acquired from hospital and primary care clinical records. Patient variables were age, sex, American Society of Anesthesiologists (ASA) score, and comorbid conditions. Patients with severe comorbidities were those with an ASA score of III-IV. Surgery-related variables were surgical procedure (right colectomy, left colectomy, segmental splenic flexure resection, total colectomy, low anterior resection, abdominoperineal resection), surgical approach (laparoscopic or open surgery), duration of operation, anastomosis, and diverting stoma. Tumor variables were tumor location, TNM classification, stage, and grade of differentiation.

### Study endpoints and outcome variables

The study endpoint was the impact of age on short-term postoperative results. Outcome variables were complications and mortality during 30 days after the intervention, comparing patient cohorts according to the age-specific cut-off point, including analysis of possible risk factors for postoperative adverse events or mortality. The variable *Any complication* was defined as any deviation from the normal postoperative course. Adverse outcomes were divided into medical and surgical complications. Clavien-Dindo classification was used to stratify postoperative complications (severe complications were those with a score ≥ III).

### Ethics

The study was approved by the local Research Ethics Committee. Informed consent was waived because of the retrospective nature of the study, and the analysis used anonymous clinical data.

### Statistical analysis

A descriptive analysis of each variable of the sample was carried out. The normality of the variables was determined by graphic methods. The description of the series was conducted according to age groups. Quantitative variables were expressed as median and range and qualitative variables as percentages. The ASA score was dichotomized to assess the risk factors of the outcome variables. Cut-off points were determined with ROC curves, considering the maximum sensibility and specificity value. Propensity score matching (PSM) was used to minimize potential selection bias. The cohort of older patients was matched to the younger cohort with a ratio 1:1. The confounding variables to calculate the PSM were sex, ASA score, tumor location, surgical procedure, laparoscopic surgery, duration of operation, and diverting stoma. Logistic regression without substitution as an estimation and nearest-neighbor pairing algorithm was performed, using 0.2 of the logarithm of the PSM standard deviation as the caliber (Supplementary File 1). After the PSM, Fisher’s exact test or *χ*^2^ tests were used to find possible differences between qualitative variables, while the Mann–Whitney *U* test was used for quantitative variables. Multivariable analysis with logistic binary regression was conducted to identify independent risk factors for postoperative complications or mortality. Internal validation of the model was performed. External validation was conducted with a sample division validation technique that randomly assigned patients into two subgroups. The model was performed with the training subset, which was 70% of the sample randomly selected, and the test subset was the remaining 30%. ROC curves and forest plots were obtained from the model. Finally, a nomogram was built according to the validated model. *P* value < 0.05 was considered statistically significant. Statistical analysis was performed using IBM SPSS Statistics for Macintosh, version 25 (IBM Corp., Armonk, N.Y., USA) and R Core Team, 2020 (R Foundation for Statistical Computing, Vienna, Austria).

## Results

### Descriptive analysis

A total of 1486 patients diagnosed with CCR were included in the study across a period of 10 years. Median patient age was 71.0 years (range: 31–95 years). Two different significant age-specific cut-off points were obtained by analyzing the influence of age on postoperative outcomes: 75 years for postoperative complications and 80 years for postoperative mortality. Patients were therefore stratified up to age 75 for sample description and analysis of complications and clustered according to age 80 for mortality assessment. Patients’ characteristics and surgery details are outlined in Tables 1 and 2 by age group. Patients aged over 75 years presented with comorbidities more frequently than non-older subjects. The tumor was more frequently located in the rectum in patients under 75 years (43.3%), whereas the ascending and transverse colon was the most frequent tumor location in the over-75s cohort (45.7%, *p* < 0.001). Consequently, the non-older group predominantly underwent low anterior resection of the rectum (36.1%), while in the older the main intervention was right colectomy (44.8%, *p* < 0.001). Anastomosis and diverting stoma were more frequently performed in the under-75s patient subset (89.4% vs. 84.8%; *p* < 0.001 and 20.1% vs. 9.8%, *p* < 0.001; respectively). Regarding tumor staging, stages II and III were more common among older patients. Given the significant differences found between the two cohorts, PSM was performed and two completely comparable groups of 438 patients were obtained.

**Table 1:** Patient characteristics by age group before and after propensity score matching

	Before propensity score matching			After propensity score matching		
Variable	Age < 75 yr. (*n* = 906)	Age ≥ 75 yr. (*n* = 580)		Age < 75 yr. (*n* = 438)	Age ≥ 75 yr. (*n* = 438)	
	Value	Value	*p*	Value	Value	*p*
*Age (years)*	64.5 (31–74)	80.0 (75–95)	** < 0.001**	64.7 (34–74)	79.0 (75–95)	** < 0.001**
*Sex*						
Male Female	551 (60.8) 355 (39.2)	318 (54.8) 262 (45.2)	**0.023**	264 (60.3) 174 (39.7)	245 (55.9) 193 (44.1)	0.218
*ASA score*			**< 0.001**			0.928
I	100 (11.0)	12 (2.1)		10 (2.3)	12 (2.7)	
II	509 (56.2)	184 (31.7)		178 (40.6)	183 (41.8)	
III	282 (31.1)	358 (61.7)		236 (53.9)	231 (52.7)	
IV	15 (1.7)	26 (4.5)		14 (3.2)	12 (2.7)	
*Comorbid conditions*						
Myocardial infarction	35 (3.9)	36 (6.2)	**0.046**	25 (5.7)	22 (5.0)	0.765
Congestive heart failure	17 (1.9)	45 (7.8)	** < 0.001**	13 (3.0)	30 (6.8)	0.012
Peripheral vascular disease	24 (2.6)	13 (2.2)	0.734	14 (3.2)	12 (2.7)	0.843
Cerebrovascular disease	27 (3.0)	38 (6.6)	**0.002**	18 (4.1)	28 (6.4)	0.172
Dementia	4 (0.4)	40 (6.9)	** < 0.001**	3 (0.7)	26 (5.9)	** < 0.001**
Chronic pulmonary disease	107 (11.8)	88 (15.2)	0.070	58 (13.2)	65 (14.8)	0.560
Peptic ulcer disease	22 (2.4)	13 (2.2)	0.863	15 (3.4)	11 (2.5)	0.551
Mild liver disease	41 (4.5)	14 (2.4)	**0.035**	21 (4.8)	11 (2.5)	0.104
Diabetes without chronic complication	194 (21.4)	171 (29.5)	** < 0.001**	122 (27.9)	127 (29.0)	0.765
Diabetes with chronic complication	5 (0.6)	3 (0.5)	1.000	3 (0.7)	2 (0.5)	1.000
Renal disease	28 (3.1)	49 (8.4)	** < 0.001**	24 (5.5)	34 (7.8)	0.221
Severe liver disease	15 (1.7)	11 (1.9)	0.840	10 (2.3)	8 (1.8)	0.813
*Tumor location*			**< 0.001**			0.769
Right and transverse colon	236 (26.0)	265 (45.7)		161 (36.8)	151 (34.5)	
Left and sigmoid colon	274 (30.2)	158 (27.2)		128 (29.2)	142 (32.4)	
Upper rectum	140 (15.5)	57 (9.8)		55 (12.6)	55 (12.6)	
Low rectum	252 (27.8)	58 (10.0)		94 (21.5)	90 (20.5)	

Statistics presented as median (min–max) or *n* (%). *p-values:* Mann–Whitney test, Pearson’s *χ*^2^ test, Fisher´s exact test

*ASA* American Society of Anesthesiologists

Boldface was used to highlight those significative *p*-values (lower than 0.05)

**Table 2:** Characteristics of surgery and histopathologic findings by age group before and after propensity score matching

	Before propensity score matching			After propensity score matching		
Variable	Age < 75 yr. (*n* = 906)	Age ≥ 75 yr. (*n* = 580)		Age < 75 yr. (*n* = 438)	Age ≥ 75 yr. (*n* = 438)	
	Value	Value	*p*	Value	value	*p*
*Surgical procedure*			**< 0.001**			0.902
Right colectomy	218 (24.1)	260 (44.8)		150 (34.2)	147 (33.6)	
Left colectomy	247 (27.3)	126 (21.7)		106 (24.2)	111 (25.3)	
Segmental splenic flexure resection	7 (0.8)	8 (1.4)		7 (1.6)	5 (1.1)	
Total colectomy	41 (4.5)	29 (5.0)		20 (4.6)	24 (5.5)	
Low anterior resection	327 (36.1)	125 (21.6)		128 (29.2)	119 (27.2)	
Abdominoperineal resection	66 (7.3)	32 (5.5)		27 (6.2)	32 (7.3)	
*Laparoscopic surgery*	417 (46.0)	264 (45.5)	0.873	197 (45.0)	205 (46.8)	0.635
*Duration of operation (min.)*	180 (50–600)	150 (47–520)	** < 0.001**	160 (50–600)	150 (47–520)	0.112
*Anastomosis*	810 (89.4)	492 (84.8)	**0.010**	388 (88.6)	372 (84.9)	0.135
*Diverting stoma*	182 (20.1)	57 (9.8)	** < 0.001**	63 (14.4)	51 (11.6)	0.269
*Neoadjuvant treatment for rectal cancer*	165 (18.2)	66 (11.4)	** < 0.001**	51 (11.6)	46 (10.5)	0.789
*Local invasion (AJCC)*			**< 0.001**			0.431
pT1	159 (17.5)	68 (11.7)		78 (17.8)	62 (14.2)	
pT2	201 (22.2)	104 (17.9)		84 (19.2)	91 (20.8)	
pT3	423 (46.7)	307 (52.9)		208 (47.5)	222 (50.7)	
pT4	123 (13.6)	101 (17.4)		68 (15.5)	63 (14.4)	
*Lymph node metastases (AJCC)*			0.344			0.344
pN0	631 (69.6)	383 (66.0)		305 (69.6)	285 (65.1)	
pN1	199 (22.0)	143 (24.7)		99 (22.6)	112 (25.6)	
pN2	76 (8.4)	54 (9.3)		34 (7.8)	41 (9.4)	
*Tumor stage (AJCC)*			**< 0.001**			0.342
I	306 (33.8)	142 (24.5)		137 (31.3)	125 (28.5)	
II	326 (36.0)	239 (41.2)		168 (38.4)	160 (36.5)	
III	274 (30.2)	199 (34.3)		133 (30.4)	153 (34.9)	
*Grade of tumor differentiation*			0.074			0.613
High	179 (19.8)	114 (19.7)		91 (20.8)	89 (20.3)	
Moderate	685 (75.6)	437 (75.3)		323 (73.7)	331 (75.6)	
Low	29 (5.0)	42 (4.6)		24 (5.5)	18 (4.1)	

Statistics presented as median (min–max) or *n* (%). *p-values:* Mann–Whitney test, Pearson’s *χ*^2^ test, Fisher’s exact test

*AJCC* American Joint Committee on Cancer, 8th edition (2018)

Boldface was used to highlight those significative *p*-values (lower than 0.05)

### Surgery outcomes

In the cohort of non-older patients, a total of 263 patients (29.0%) presented postoperative complications during the 30 postoperative days, while 39.0% of the older patients suffered any postoperative adverse event (*p* < 0.001). Table 3 shows surgery outcomes. After matching the two age cohorts, the only differences found between them were in respiratory and cardiac complications. Only cases with anastomosis were included in the analysis of anastomotic failure, without between-group differences. Postoperative transfusion was needed more frequently in patients aged ≥ 75 years. According to the Clavien-Dindo classification, older patients suffered severe complications (≥ III) more often than the younger subset (16.2% vs 11.9%, *p* < 0.001). The postoperative mortality rate was 2.5% across the whole series. Patients aged ≥ 80 years presented a higher mortality rate during the first 30 postoperative days than those aged under that cutoff (5.3% vs. 0.8%, respectively; *p* < 0.001) and after matching the two cohorts, these differences remained (*p* = 0.024).

**Table 3:** Surgery outcomes before and after propensity score matching

	Before propensity score matching			After propensity score matching		
Variable	Age < 75 yr. (*n* = 906)	Age ≥ 75 yr. (*n* = 580)		Age < 75 yr. (*n* = 438)	Age ≥ 75 yr. (*n* = 438)	
	Value	Value	*p*	Value	Value	*p*
*Length of stay (days)*	8 (1–311)	8 (1–89)	** < 0.001**	8 (1–154)	8 (1–89)	**0.021**
*Any complication during the episode (30 days)*	263 (29.0)	226 (39.0)	** < 0.001**	138 (31.5)	165 (37.7)	0.065
*Medical complications during the episode (30 days)*	68 (7.5)	95 (16.4)	**< 0.001**	42 (9.6)	72 (16.4)	**0.003**
Respiratory complications	30 (3.3)	64 (11.0)	** < 0.001**	20 (4.6)	46 (10.5)	** < 0.001**
Cardiac complications	17 (1.9)	35 (6.0)	** < 0.001**	12 (2.7)	26 (5.9)	**0.030**
Urinary complications	38 (4.2)	28 (4.8)	0.606	20 (4.6)	23 (5.3)	0.755
Cerebrovascular accident	2 (0.2)	3 (0.5)	0.384	2 (0.5)	1 (0.2)	1.000
Upper gastrointestinal bleeding	1 (0.1)	3 (0.5)	0.306	1 (0.2)	3 (0.7)	0.624
*Surgical complications during the episode (30 days)*	229 (25.3)	177 (30.5)	**0.027**	117 (26.7)	131 (29.9)	0.330
Surgical site infection	126 (13.9)	89 (15.3)	0.450	69 (15.8)	67 (15.3)	0.926
Superficial	28 (3.1)	15 (2.6)	0.636	18 (4.1)	14 (3.2)	0.590
Deep	7 (0.8)	4 (0.7)	1.000	4 (0.9)	3 (0.7)	1.000
Organ space	42 (4.6)	29 (5.0)	0.803	19 (4.3)	20 (4.6)	1.000
Ileus	76 (8.4)	66 (11.4)	0.058	34 (7.8)	44 (10.0)	0.286
Anastomotic leak	57 (6.3)	44 (7.6)	0.343	30 (6.8)	34 (7.8)	0.697
Enterocutaneous fistula	17 (1.9)	5 (0.9)	0.128	3 (0.7)	5 (1.1)	0.725
Wound disruption	14 (1.5)	21 (3.6)	**0.013**	10 (2.3)	15 (3.4)	0.418
Postoperative bleeding	6 (0.7)	8 (1.4)	0.177	2 (0.5)	6 (1.4)	0.287
Intestinal ischaemia	7 (0.8)	5 (0.9)	1.000	4 (0.9)	5 (1.1)	1.000
Stoma complications	5 (0.6)	7 (1.2)	0.234	4 (0.9)	7 (1.6)	0.546
Intestinal perforation	3 (0.3)	2 (0.3)	1.000	2 (0.5)	1 (0.2)	1.000
Iatrogenic urinary lesions	3 (0.3)	0 (0.0)	0.524	2 (0.5)	0 (0.0)	0.499
*Perioperative transfusion*	61 (6.7)	71 (12.2)	** < 0.001**	39 (8.9)	51 (11.6)	0.221
*Postoperative transfusion*	96 (10.6)	108 (18.6)	** < 0.001**	53 (12.1)	80 (18.3)	**0.014**
*Reoperation*	71 (7.8)	62 (10.7)	0.063	39 (8.9)	47 (10.7)	0.427
*Readmission*	27 (3.0)	6 (1.0)	**0.012**	13 (3.0)	5 (1.1)	0.093
*Clavien-Dindo classification*			**< 0.001**			**0.015**
0	643 (71.0)	354 (61.0)		300 (68.5)	273 (62.3)	
I	60 (6.6)	43 (7.4)		38 (8.7)	36 (8.2)	
II	89 (9.8)	58 (10.0)		38 (8.7)	41 (9.4)	
IIIa	28 (3.1)	12 (2.1)		13 (3.0)	10 (2.3)	
IIIb	45 (5.0)	32 (5.5)		23 (5.3)	22 (5.0)	
IVa	26 (2.9)	37 (6.4)		18 (4.1)	31 (7.1)	
IVb	8 (0.9)	13 (2.2)		4 (0.9)	10 (2.3)	

Statistics presented as median (min–max) or *n* (%)

*p-values*: Mann–Whitney test, Pearson’s *χ*^2^ test, Fisher’s exact test

Boldface was used to highlight those significative *p*-values (lower than 0.05)

### Risk factors for postoperative complications

**Fig. 1:**
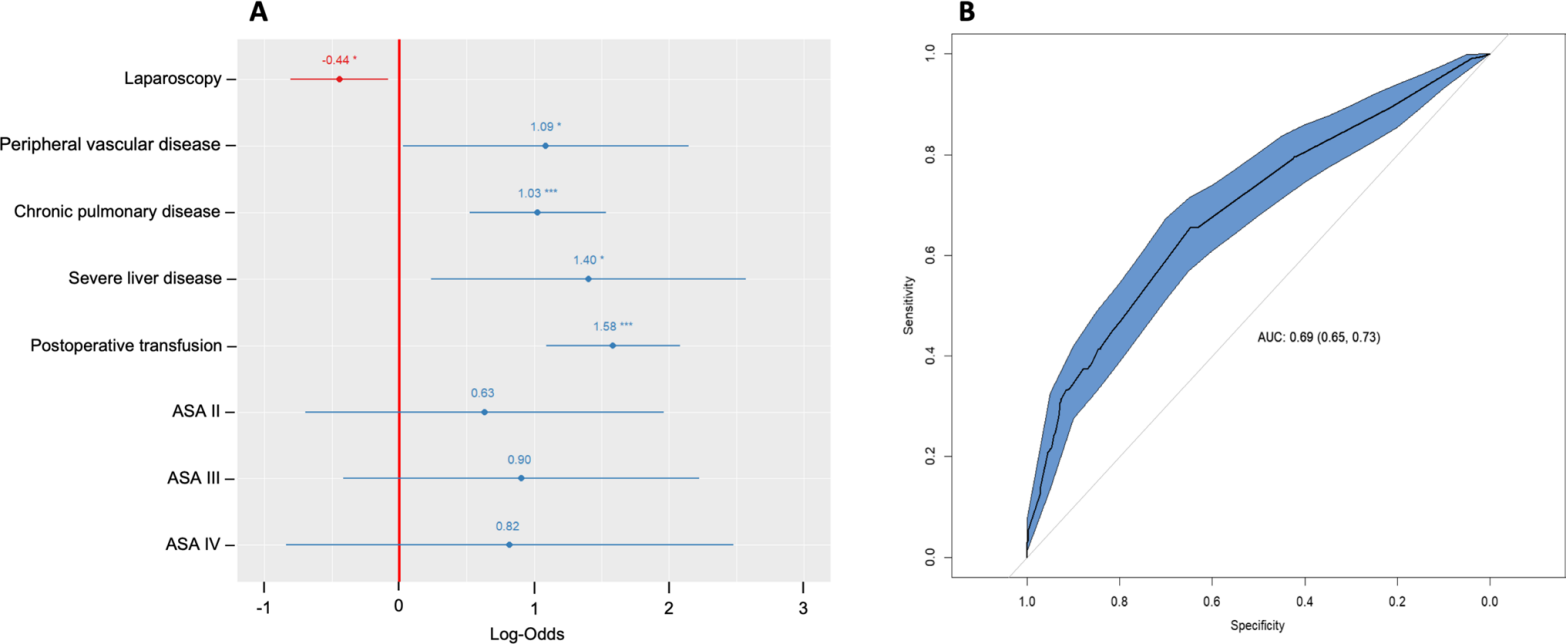
Multivariable analysis model for postoperative complications. **A**: Forest plot of independent risk factors. **B**: Receiver operating characteristic curves of the model

We conducted univariable and multivariable analysis of factors associated with postoperative complications. As depicted in the forest plot (Fig. 1), binary logistic regression revealed independent risk factors for presenting any complication to be peripheral vascular disease, chronic pulmonary disease, severe liver disease, and postoperative transfusion. However, the laparoscopic approach was an independent factor predicting a lower postoperative complication rate. All these factors showed a variance inflation factor under 1.5. The model had an area under the curve of 0.69 (*IC* 95% = 0.65–0.73) and 70.3% accuracy. Age was not an independent risk factor for postoperative complications and moreover showed no association with surgical site infection (*p* = 0.181), anastomotic leak (*p* = 0.636), or reoperation rate (*p* = 0.195).

### Risk factors for postoperative mortality

**Fig. 2:**
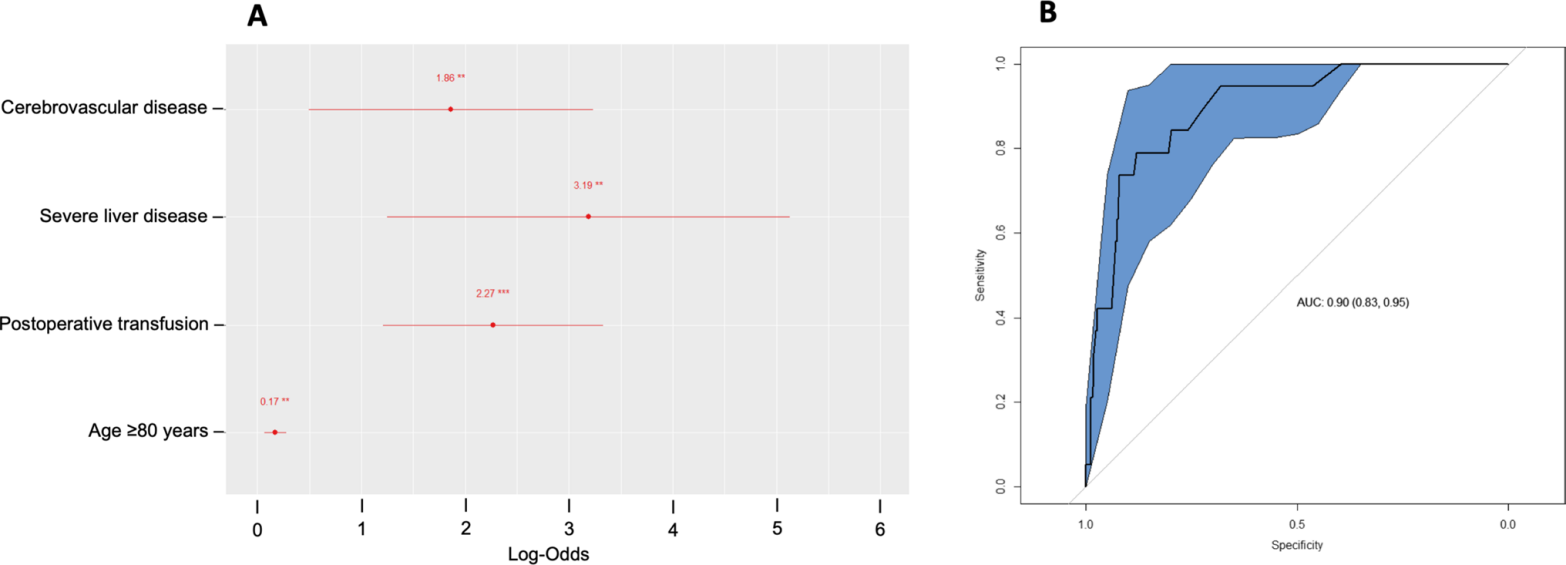
Multivariable analysis model for 30-day mortality. **A**: Forest plot of independent risk factors. **B**: Receiver operating characteristic curves of the model

The forest plot of Fig. 2 represents independent risk factors for postoperative mortality obtained from multivariable analysis with binary logistic regression: age ≥ 80 years, cerebrovascular disease, severe liver disease, and postoperative transfusion. Variance inflation factor was lower than 1.3 in all factors. The model presented an area under the curve of 0.90 (*IC* 95% = 0.83–0.95) and an accuracy of 93.9%.

### Nomograms

**Fig. 3:**
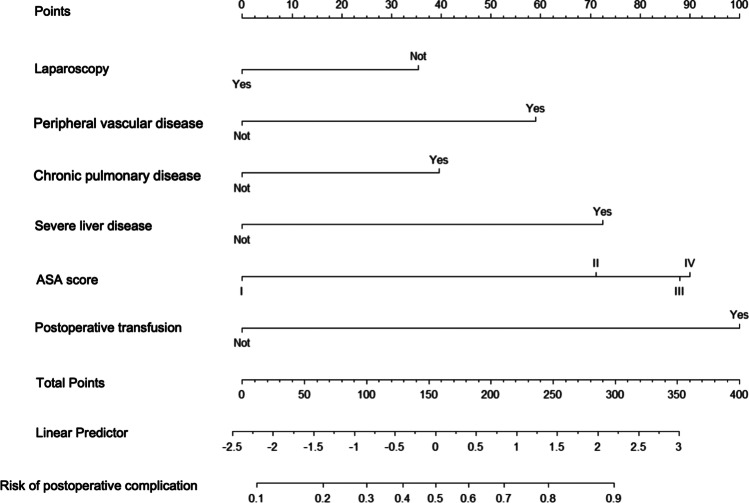
Prognostic nomogram of postoperative complications after colorectal cancer surgery

**Fig. 4:**
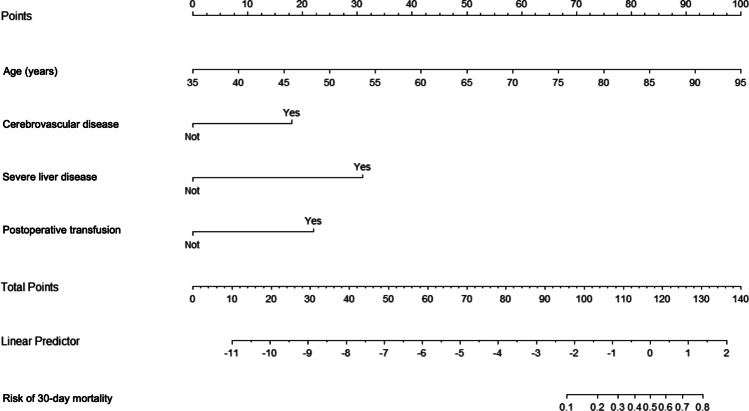
Prognostic nomogram of 30-day mortality after colorectal cancer surgery

Two nomograms were constructed to predict the risk of complications and mortality during the postoperative period (Figs. 3, 4). The value of each risk factor is obtained from the upper percentile line, and their sum gives an overall score indicating the probability of postoperative complications or 30-day mortality in the risk line at the bottom. The prognostic nomogram of postoperative complications after colorectal cancer surgery showed an accuracy of 68.4% with an area under the ROC curve of 70%, and the prognostic nomogram of 30-day mortality was able to predict postoperative mortality with an accuracy of 90.2% and an area under the ROC curve of 91%.

## Discussion

This is one of the few studies to focus on analysis of independent risk factors for postoperative complications and mortality, employing a substantial sample size with detailed perioperative data and providing a novel nomogram to predict short-term outcomes.

To establish two age groups, the age-specific cut-off point of ≥ 75 years was fitted for the present series, thus providing non-arbitrary sample stratification. In most previous published studies, there is great variability between the age thresholds selected for postoperative outcomes in older patients; they are usually standard cut-off points, not representing inflection points in the series and without medical or biological evidence to support the choice. Some authors also found that age ≥ 75 years could be an optimal cut-off, and age has also been stated as a significant risk factor for postoperative complications in colorectal surgery [12]. After analyzing data on physical and psychological health in the older, the Japanese Geriatrics Society proposed that elderly should be defined as those aged 75 years and older [23]. Nevertheless, other authors classified older patients into three groups: youngest-old (65 to 74 years), middle-old (75 to 84 years) and oldest-old (≥ 85 years) [24].

Comparing the two age groups, older patients had a greater number of comorbidities, which were more also severe. Improvements in perioperative multidisciplinary care have made colorectal surgery feasible in the older despite the fact that they frequently present with serious comorbidities [4–6]. Similar to other available studies, in the aged cohort, the tumor was more frequently located in the ascending colon, resulting in a higher ratio of right colectomies [6, 16]. As the two subsets differed in their baseline features, PSM was conducted to obtain two homogeneous groups in order to compare postoperative outcomes. Note that all study patients included received the same perioperative bundle of enhanced recovery after surgery protocols, regardless of age.

Similarly to other authors, we found no differences between older and younger patients in postoperative complication rates, including anastomotic leak [6, 13]. These results support that in patients eligible for colorectal resection, a primary anastomosis can be performed safely without excess risk. A recent systematic review and meta-analysis conducted by Hoshino et al,. focusing on the outcomes of laparoscopic surgery for CCR in older patients, reported slightly higher incidence of postoperative complications in the older, but without differences in anastomotic leak or mortality rates [10].

Our findings revealed that severe postoperative complications were mainly due to worsening of previous comorbidities. Cardiopulmonary complications were more frequent among patients aged ≥ 75 years. Chan et al. also reported pneumonia with respiratory failure as the most common postoperative complication and the leading cause of mortality [17]. In a study of over 1200 CCR patients aged ≥ 85 years undergoing surgical resection, Verweij et al. found high rates of cardiopulmonary complications and excess mortality, particularly in the first year after surgery [11].

The mortality rate for older patients during the postoperative period was 5.3%, in line with outcomes obtained in other studies on octogenarians (2%–13%) and nonagenarians (2%–20%) [4, 5, 9, 11–16]. Although older patients may present more comorbidities, several studies found no differences in short-term postoperative reoperations or mortality after colorectal surgery [17–19]. Improvements in mortality rates are likely because of advances in perioperative care, safe standardized minimally invasive procedures and better patient selection for surgery. In our experience, although colorectal resection did not involve higher postoperative complication rates in older patients, it did entail higher mortality rates, predominantly in patients with associated comorbidities. Prehabilitation programs could help to optimize preoperative patient status, minimize postoperative risks, and improve surgical outcomes. Furthermore, aged patients without concurrent diseases can be successfully treated by curative-intent surgery. Comorbidities may therefore have more impact on postoperative outcomes than age itself.

Age has long been considered among the predominant risk factors for postoperative complications, but essentially due to an increased number of comorbid conditions and worse functional status [4, 11, 12]. Likewise, multivariable analysis revealed that several comorbidities, but not age, were independent predictors of postoperative complications. Moreover, age did not present any association with surgical site infection, anastomotic leak, or with reoperation rate. These findings are consistent with those obtained from other large series, where age was not predictive of in-hospital complications or mortality, suggesting that other conditions may impact more significantly in surgical outcomes [8, 12, 14, 19, 20, 25]. Therefore, it would be more appropriate to consider a frailty index rather than age in preoperative decision-making. Identification of predictors for surgical complications in elderly frail patients could be useful to implement further optimization bundles before major surgery.

Chronic pulmonary disease was an independent risk factor for postoperative adverse events. In other studies, preoperative cardiopulmonary function was determinant in postoperative outcomes [11, 17]. Respiratory physiotherapy is a good measure to incorporate in perioperative care for older patients, given that it could decrease incidence of postoperative pulmonary complications and 30-day mortality [26].

Severe liver disease is a serious comorbidity and was found to be independently associated with adverse postoperative outcomes. Similarly, a recent meta-analysis concluded that pre-existing liver cirrhosis was associated with higher postoperative major complication and mortality rates following CRC surgery [27]. One reason for this could be that abnormal liver metabolism leads to hepatic coagulopathy, lower albumin levels, reduced drug metabolism, and weakened immune function, increasing postoperative adverse events.

Laparoscopic surgery is safe in older patients, and moreover, postoperative complications including wound infection, ileus, and pneumonia are less frequent than in open surgery [8, 12, 18, 19, 28]. In the present series, laparoscopic approach was found to be independently associated with a lower postoperative complication rate. Similarly, a Dutch population-based study found that compared with open surgery, laparoscopic surgery was independently associated with a lower risk of cardiopulmonary complications and reduced risk of postoperative mortality in elective CCR surgery [21]. Older patients could benefit from laparoscopic surgery despite their limited life expectancy and comorbidities.

Undoubtedly one of the most interesting aspects of our study is the determination of factors influencing postoperative death. In recent years, various prognostic factors for 30-day postoperative mortality have been outlined in older patients, such as age ≥ 85 years, anemia, ASA score IV, and palliative cancer surgery [13]. We found that age ≥ 80 years, cerebrovascular disease, severe liver disease, and need for postoperative transfusion increased the risk of 30-day mortality. Interestingly, advanced age was not predictive of complications, but was revealed as a predictor for postoperative mortality. A possible explanation could be that although older patients present a similar postoperative complication rate to younger ones, recovery is more hazardous in the former group due to their limited physiological reserve, which could entail a higher risk of mortality. These outcomes are in line with those obtained by Youl et al. in a population-based study in Australia which analyzed postoperative outcomes in 18,339 patients aged over 65 years diagnosed with CRC. Among other factors such as advanced tumor stage, open procedure, and emergency surgery, age ≥ 75 years was found to be independently related with an increased risk of postoperative death [12]. Other studies have also concluded that comorbidities were the main factors influencing mortality after surgery, but age itself was not [14, 17, 25].

Another aspect frequently associated with worse postoperative complications potentially leading to increased mortality is the need for postoperative transfusion. As expected, therefore, blood transfusion was revealed as a prognostic factor for 30-day complications and mortality, consistent with the results reported in other studies [16]. Postoperative transfusion may reveal intraoperative bleeding. However, in the present series, the main indication for transfusion was the worsening of preoperative preexisting anemia. Many studies reported worse outcomes when blood transfusion was needed during the postoperative period, particularly in elderly patients. Some authors found that perioperative blood transfusion was a very good predictor of postoperative mortality [29, 30]. Older patients have limited physiological reserve, making this subset of patients especially vulnerable to the consequences of anemia, therefore preoperative optimization of hemoglobin level should be recommended.

Similarly, emergent surgery is known to negatively affect surgical outcomes and has been widely proposed as a predictor of postoperative mortality in older patients [4, 11–14, 17]. In the present series, however, we included elective surgery only to diminish confounding factors in the analysis and avoid heterogeneity between groups.

The nomograms constructed in the present study are in line with the few that have previously been published. As in Kiran et al., our model was built with a 70% randomly selected study population, and the remaining 30% used to validate it. This ratio was used to avoid overfitting the model. In the multicenter national study conducted by Anaco Study Group, however, the ratio was 60/40 [31, 32]. The models presented similar areas under the curve and share some risk factors. The predictive novel nomograms developed in the present study confirm that prior severe comorbid conditions are the main factors in postoperative short-term outcomes. The nomograms presented herein are useful tools in our setting, as they easily provide individualized risk prediction of postoperative complications or mortality, can help clinicians in preoperative evaluation by providing accurate information about postoperative risks, and could facilitate enhanced, tailored multidisciplinary care to minimize complications.

The study included a relatively large sample with non-arbitrary age cut-off points and two homogeneous patient groups obtained through PSM that received the same perioperative care. The prediction model constructed obtained high accuracy and satisfactory internal and external validation and was presented in the form of a nomogram to facilitate its application by clinicians in outpatient clinics. Nonetheless, this study has some limitations, arising from its observational and retrospective design at a single institution. Patients diagnosed with rectal cancer were included in the study because after the PSM, this subgroup of patients was equally distributed between the two age groups; however, this could be a potential source of bias given that rectal surgery is more complex and time consuming than colon surgery. Data about performance status, frailty, sarcopenia, or nutritional status were not recorded, so accurate information about the functional status of the patients was limited.

## Conclusion

Patients aged over 75 years with CCR who underwent oncologic surgery presented a similar complication rate but higher mortality rate than younger patients during the postoperative period. Patients with severe comorbidities (peripheral vascular disease, chronic pulmonary disease, or severe liver disease) should be informed of higher postoperative complications, regardless of age, but patients aged over 80 suffering cerebrovascular disease, or severe liver disease, or needing postoperative transfusion should be warned of a significantly increased risk of postoperative mortality. The novel nomogram proposed herein could help tailor management of patient comorbidities and target perioperative care to improve outcomes.

## Supplementary information

Below is the link to the electronic supplementary material.

Supplementary file1 Flowchart of propensity score matching of study patients. (PDF 24 KB)

## Data Availability

Data supporting the findings of this study are available from the corresponding author upon reasonable request.
