# Supplementary figures and images for: Short-term outcomes of colorectal cancer surgery in older patients: a novel nomogram predicting postoperative morbi-mortality
Source: Langenbecks Arch Surg. 2022 Sep 21;407(8):3587–97. doi: 10.1007/s00423-022-02688-1 (PMC9722849; doi:10.1007/s00423-022-02688-1)

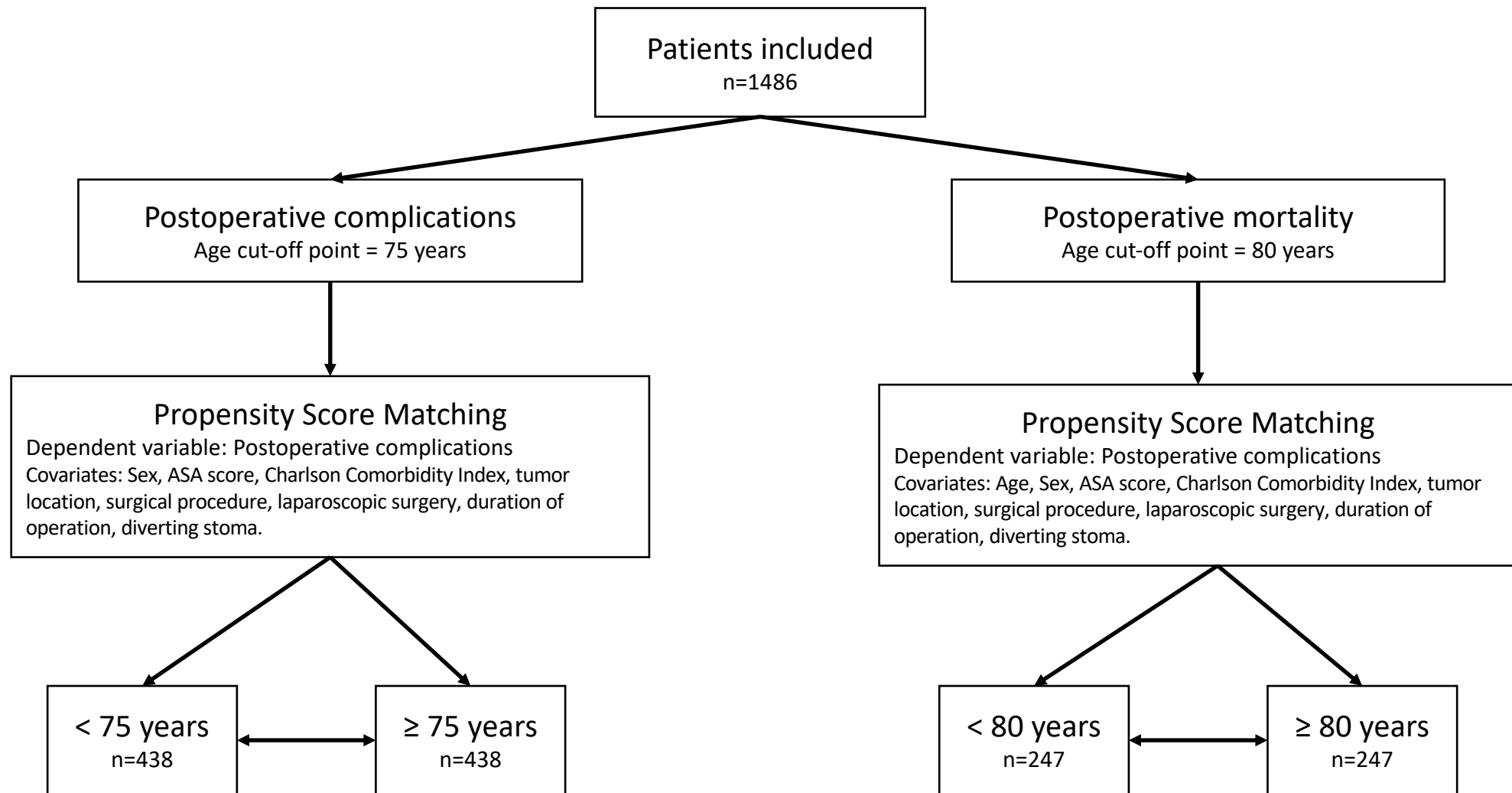

Supplement: Supplementary file 1 — Supplementary file1 Flowchart of propensity score matching of study patients. (PDF 24 KB) [file 423_2022_2688_MOESM1_ESM.pdf]
